# Acute myocardial infarction in the elderly with anomalous origin of the left coronary artery from the pulmonary artery (ALCAPA): A case report and literature review

**DOI:** 10.1097/MD.0000000000032219

**Published:** 2022-12-02

**Authors:** Mengyao Niu, Jing Zhang, Yanmin Ge, Xiaohui Hu, Zhihao Liu, Junduo Wu

**Affiliations:** a Department of Cardiology, The Second Hospital of Jilin University, Changchun, Jilin, China.

**Keywords:** acute myocardial infarction, ALCAPA, the elderly

## Abstract

Anomalous left coronary artery from the pulmonary artery (ALCAPA) is a rare congenital coronary artery malformation, with a fatality rate of 90% at 1 year of age; only 10% to 15% of patients are diagnosed in adulthood. However, elderly survivors are particularly rare. Here, we report a case of elderly ALCAPA presented with acute myocardial infarction.

**Case presentation:**

A 64-years-old female, complained of acute precordial pain in our hospital for 2 days. She was diagnosed with an acute non-ST-segment elevation myocardial infarction. Aortic angiography revealed emptiness of the left coronary sinus, and coronary angiography showed that the tortuous right coronary artery supplied blood to the left coronary artery through collateral circulation, and the contrast medium spilled from the opening of the left coronary artery. It was suspected that the left coronary artery was opened in the pulmonary artery. This finding was subsequently confirmed by coronary artery CT. The patient refused surgery to restore double coronary circulation and was administered standardized drug treatment. There was no chest pain during the 6-month follow-up.

**Conclusion:**

ALCAPA should be considered in patients with Myocardial Infarction with Non-obstructive Coronary Arteries, and surgical intervention is the first choice for such patients; However, chronic myocardial damage persists regardless of surgical treatment, prophylactic implantation of an ICD may be an important means of preventing sudden cardiac death and such patients should be followed up for a lifetime.

## 1. Introduction

Left coronary artery (LCA) malformation originating from the pulmonary artery (PA), also known as Bland-White-Garland syndrome, is a rare and dangerous congenital heart disease with a fatality rate of 90% within 1 year of age; only 10% to 15% of patients are diagnosed in adulthood.^[1]^ In this paper, we report a rare case of elderly female anomalous left coronary artery from the pulmonary artery (ALCAPA) and review the related literature.

## 2. Case report

**Figure 1.**
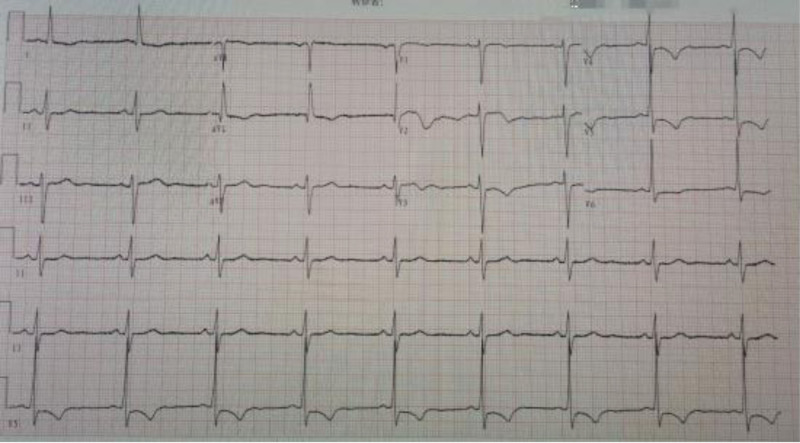
Electrocardiogram: abnormal *Q* wave in lead a VL, *T* wave inversion in lead I, a VL and V1–V6, depression of 0.1mV in ST of lead V4 and V5, and left ventricular hypertrophy.

A 64-years-old female was admitted to the hospital because of “paroxysmal precordial pain for 10 years, aggravated for 2 days” on March 10, 2022. The patient had a history of hypertension. Cardiac troponin I:0.21ng/mL (reference value range:0.01–0.023ng/mL) was examined on admission. Electrocardiogram showed an abnormal *Q* wave in lead a VL, *T* wave inversion in lead I, a VL and *V*1 to *V*6, 0.1mV’s depression in the ST of lead *V*4 and *V*5, and left ventricular hypertrophy (Fig. 1). The patient was diagnosed with “acute non-ST-segment elevation myocardial infarction” and was treated with standard drugs.

**Figure 2.**
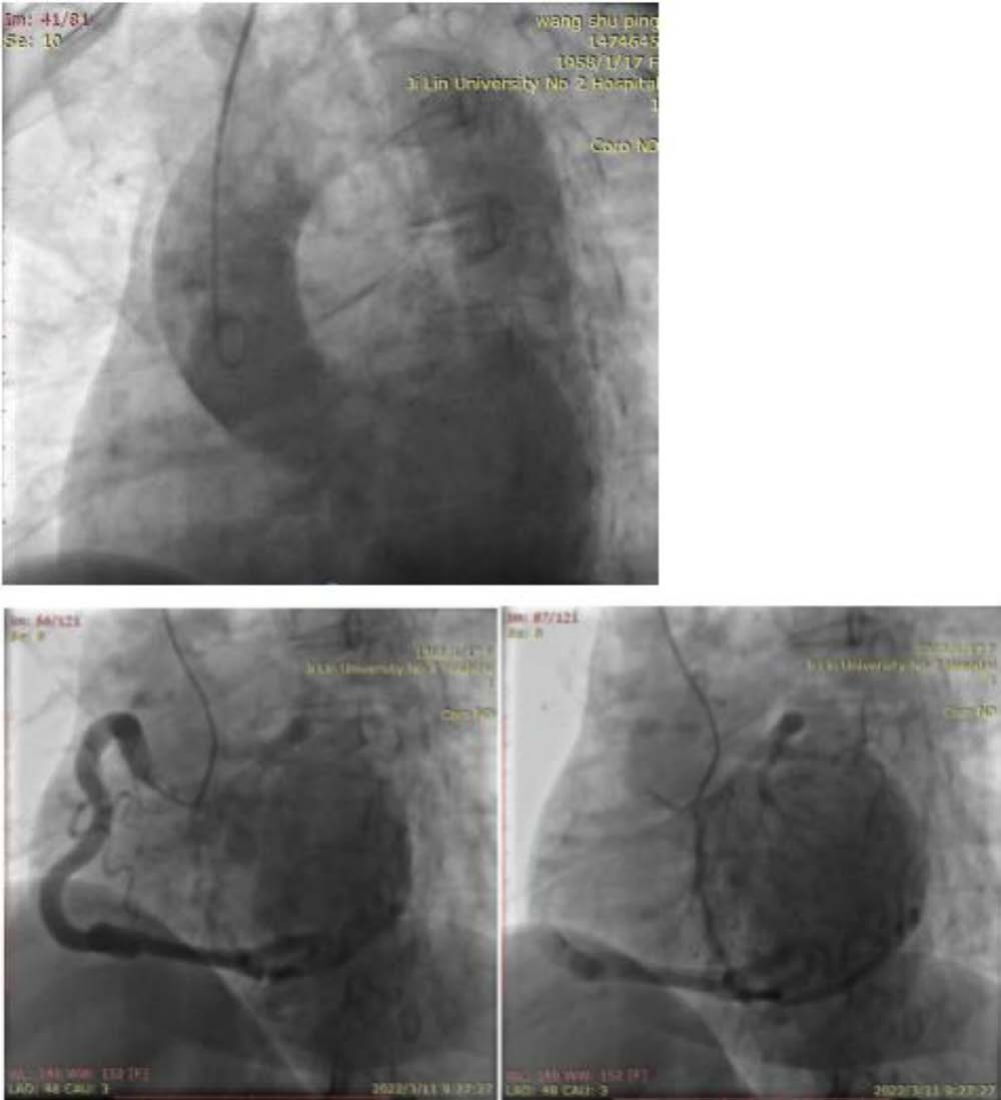
CAG:(A): no LCA opening was found in the aortic sinus by Aortic angiography. (B, C): RCA angiography found that the RCA was abnormally large, reverse blood supply to LCA through collateral circulation, and leakage of contrast medium from the opening of LCA. CAG = coronary angiography, LCA = left coronary artery, RCA = right coronary artery.

During Aortic and coronary angiography, no LCA opening was found by using pigtail catheter in the aortic sinus and the right coronary artery (RCA) was found to be abnormally large on right coronary angiography. The RCA supplied blood to the LCA through collateral circulation, and leakage of the contrast medium from the opening of the LCA could be seen, considering that it might flow to the blood vessels with lower pressure. During the operation, it was highly suspected that the opening of the LCA was in the PA, but the opening of the LCA could not be found by repeated angiography in the PA with a pigtail catheter (probably due to low pressure in the PA) (Fig. 2).

**Figure 3.**
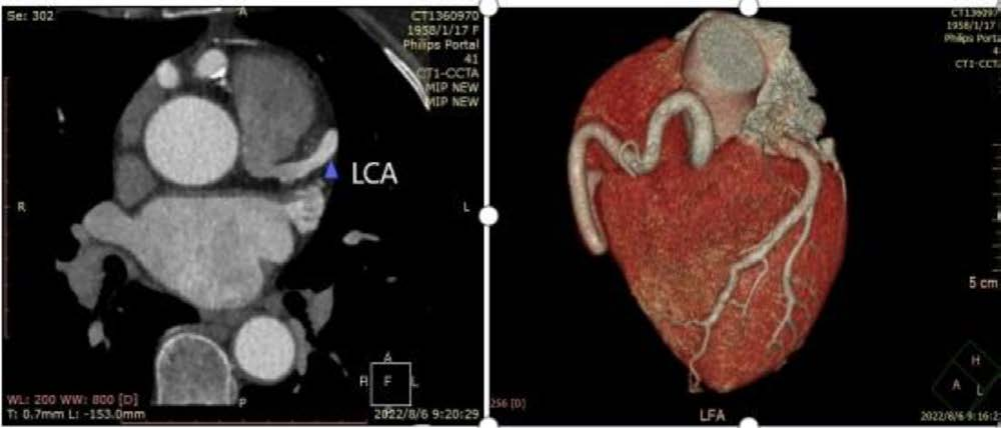
MDCT: The RCA is tortuous and thick, which originates from the aorta and The LCA originates from the pulmonary artery abnormally. LCA = left coronary artery, MDCT = multi-detector computed tomography, RCA = right coronary artery.

Multi-detector computed tomography (MDCT) confirmed this inference (Fig. 3). Surgical restoration of double coronary artery circulation was recommended, but the patient refused. Currently, standardized drug treatment is performed, and there is no significant chest pain during follow-up for 6 months.

## 3. Discussion

An elderly woman with acute chest pain was admitted to the hospital. The clinical diagnosis of ALCAPA was confirmed by coronary angiography (CAG) and MDCT, which is extremely rare in the clinic; therefore, this article mainly introduces adult ALCAPA.

ALCAPA mostly originates from the left posterior sinus at the root of the PA, which can lead to adverse events such as hypoxemia, coronary artery steal syndrome, and myocardial ischemia; the incidence of the disease is approximately 1/3,00,000, accounting for 0.25% to 0.5% of congenital heart disease. Among them, 5% of cases coexist with cardiac malformations, such as atrial septal defect, ventricular septal defect, tetralogy of Fallot, and coarctation of the aorta. According to the degree of collateral circulation between the left and right coronary arteries,^[1]^ ALCAPA is divided into infantile and adult types

Adult ALCAPA has rich collateral circulation and various clinical manifestations, ranging from asymptomatic to mitral regurgitation, exertional angina, myocardial infarction, heart failure, malignant arrhythmia, and sudden cardiac death (SCD), and approximately 90% of patients have SCD at an average age of 35.^[2]^

**Figure 4.**
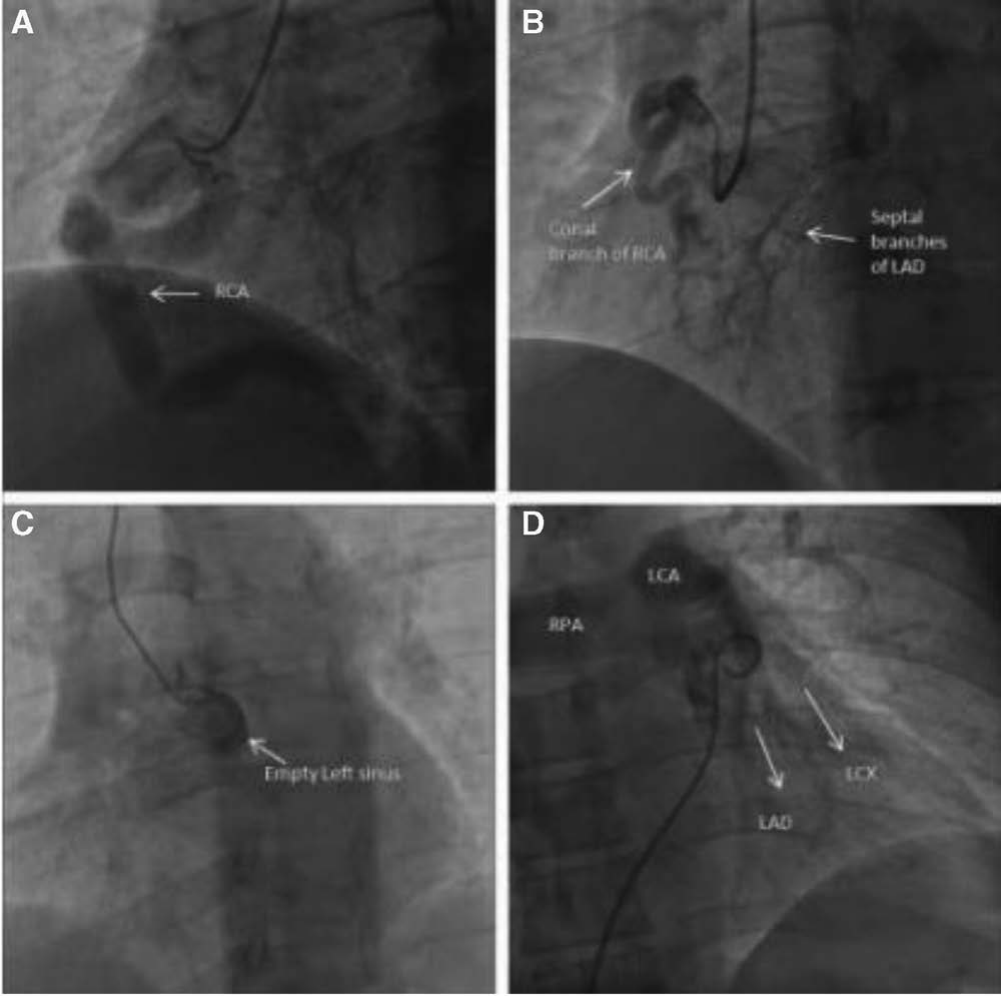
(A) CAG shows a large RCA. (B) There are a large number of collateral vessels in the right and left coronary arteries. (C) CAG showed emptiness of the left coronary sinus. (D) Pulmonary angiography showed that LCA originated from the PA.^[4]^ CAG = coronary angiography, LCA = left coronary artery, PA = pulmonary artery, RCA = right coronary artery.

Diagnosis of the disease was mainly confirmed by CAG, CT, magnetic resonance imaging (MRI) and echocardiography. The gold standard for the diagnosis of ALCAPA is CAG, which indicates that RCA is a tumor-like dilatation, the left aortic sinus is empty, and the contrast medium flows from the tortuous RCA to the LCA through collateral circulation, and then retrograde filling PA; LCA originates from the PA^[3]^ (Fig. 4). In this case, the abnormal origin of the LCA was identified using CAG.

**Figure 5.**
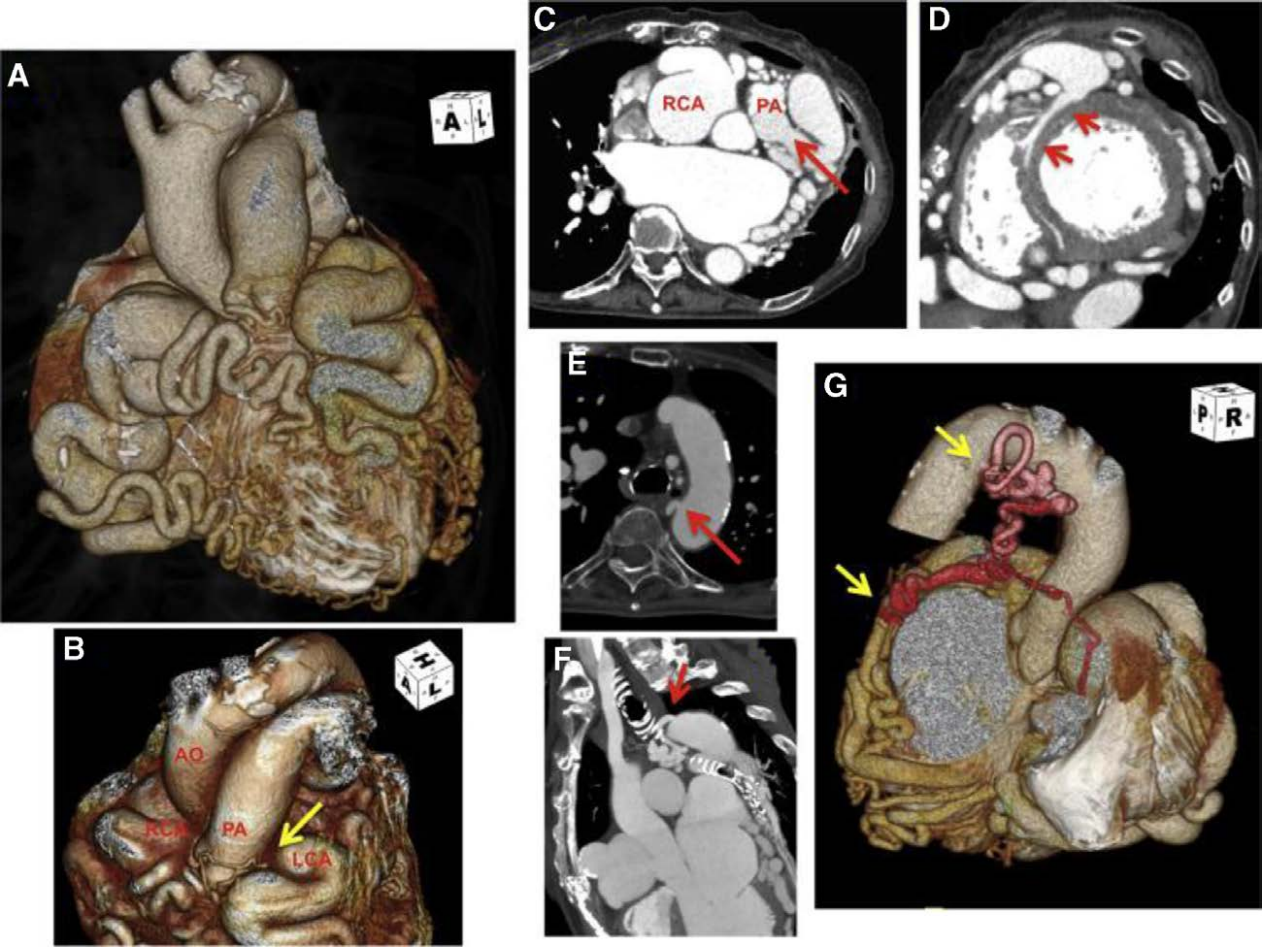
Volume-rendering (VR) images (A, B) and slice views (C) of coronary computed tomography angiography are shown. (A, B, C)Coronary MDCT showed anomalous origin of LCA from the PA (arrow), where RCA and LCA are tortuous and dilated; (C) tumor-like dilated RCA emits multiple collateral vessels (arrow), (D) Short axis slices showed markedly dilated septal branch perforating through myocardium; (E, F)the bronchial artery forms collateral branches from the aortic arch (arrow) to the left atrium and (G) supplies the left circumflex artery (arrow).^[5]^ LCA = left coronary artery, MDCT = multi-detector computed tomography, PA = pulmonary artery, RCA = right coronary artery.

**Figure 6.**
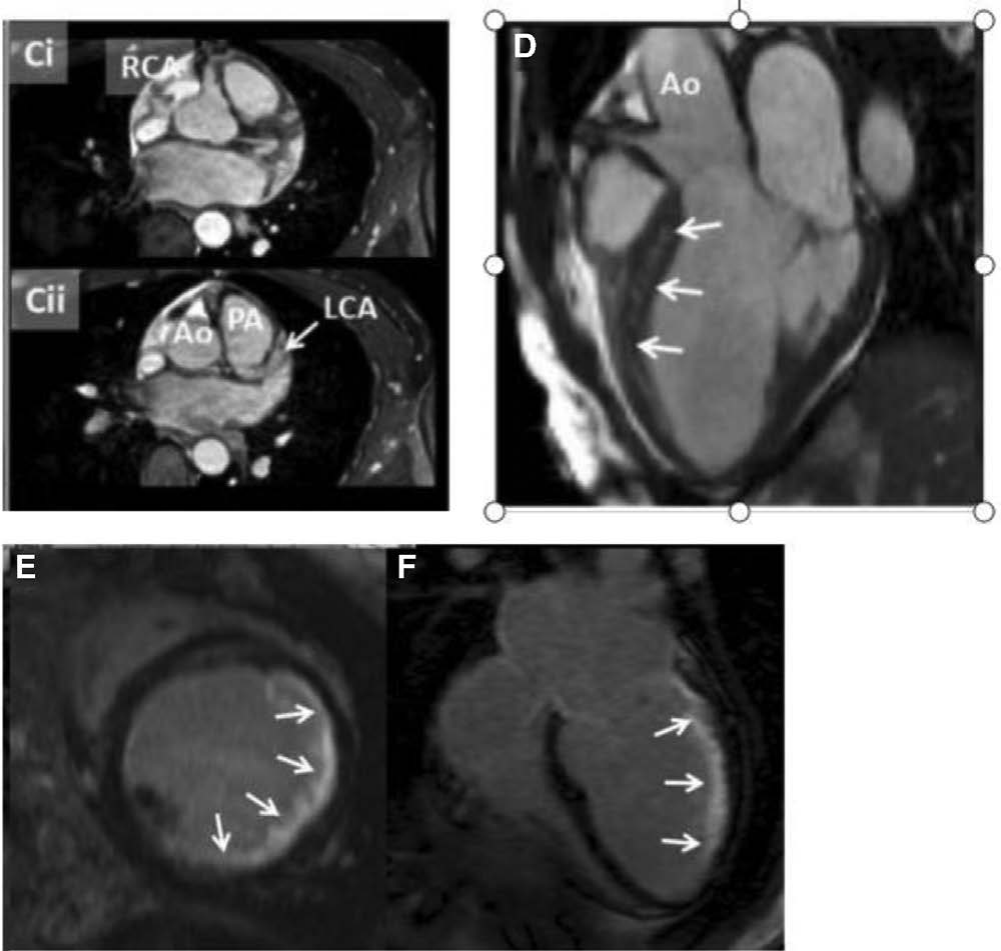
Cardiac MRI ECG and respiratory navigator-gated 3D SSFP-axial plane Ci: Dilated RCA originates from the right coronary sinus normally. Cii: LCA originates from PA anomalously. (D) Cardiac MRI-SSFP cine image 3 chamber view:The collateral vessels flowing from RCA to LCA can be seen in the septal wall. Cardiac MRI-late gadolinium images. Basal short axis (E) and 4 chamber view (F): Sub endocardial myocardial fibrosis in the lateral wall.^[6]^ LCA = left coronary artery, MRI = magnetic resonance imaging, PA = pulmonary artery, RCA = right coronary artery.

Because of the invasiveness of CAG, noninvasive ECG-gated MDCT and MRI are often adopted as the first choice for the diagnosis of ALCAPA; both have their own advantages and disadvantages. MDCT is widely used because of its high spatial resolution and rapidity; its disadvantage is that it uses ionizing radiation and is unable to evaluate blood flow (Fig. 5). MRI temporary resolution is high, and cine phase-contrast imaging can quantify blood flow from LCA to PA. Delayed enhancement can evaluate left ventricular myocardial viability; steady-state free precession (SSFP) cinematography can evaluate retrograde blood flow from the LCA to the PA, wall motion, and valvular abnormality, but it has the disadvantages of low spatial resolution and is time-consuming.(Fig. 6)^[2]^ the comparison of MDCT and MRI in the diagnosis of ALCAPA is shown in Table 1.

**Table 1:** Comparison of ECG-gated Multi-detector computed tomography and magnetic resonance imaging in the diagnosis of Anomalous Origin of the Left Coronary Artery from the Pulmonary Artery.

Image	Common Features	Exclusive Feature	Clinical significance
CT	The LCA originated from the PA, Tortuous dilatation of LCA and RCA, Collateral circulation along the epicardium or interventricular septum, Left ventricular hypertrophy and dilatation, Abnormal left ventricular wall motion	Dilated bronchial artery can be seen.	Preliminary diagnosis and postoperative follow-up
CMR		Quantification of reverse blood flow from LCA to PA, Mitral valve prolapse and regurgitation, Delayed subendocardial myocardial enhancement.	Preoperative assessment of left ventricular myocardial infarction size, abnormal wall motion, mitral valve injury and the degree of left-to-right shunt; The viability of left ventricular myocardium can also be evaluated before and after operation to determine whether asymptomatic adults can benefit from surgical treatment and whether ICD implantation is needed after operation.

LCA = left coronary artery, PA = pulmonary artery, RCA = right coronary artery.

**Figure 7.**
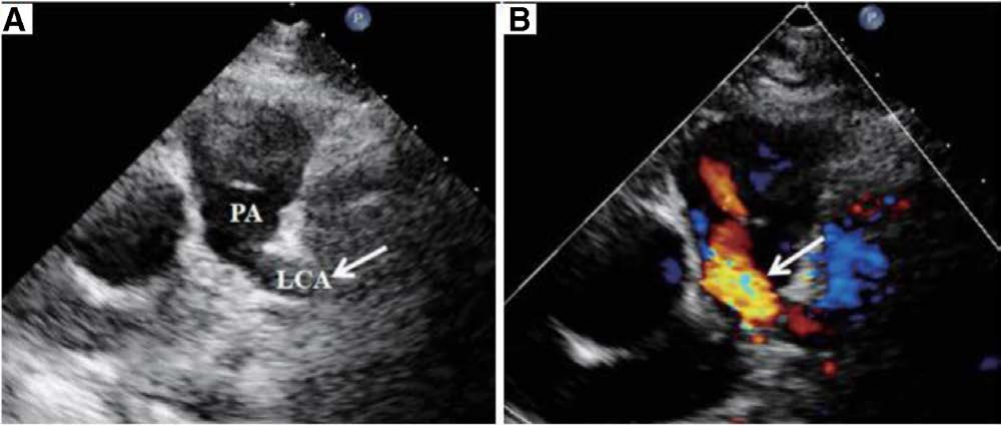
(A) Echocardiography shows the LCA originating from the posterior wall of the pulmonary trunk (arrow); (B) The blood of the LCA flows back into the PA.^[7]^ LCA = left coronary artery, PA = pulmonary artery.

**Figure 8.**
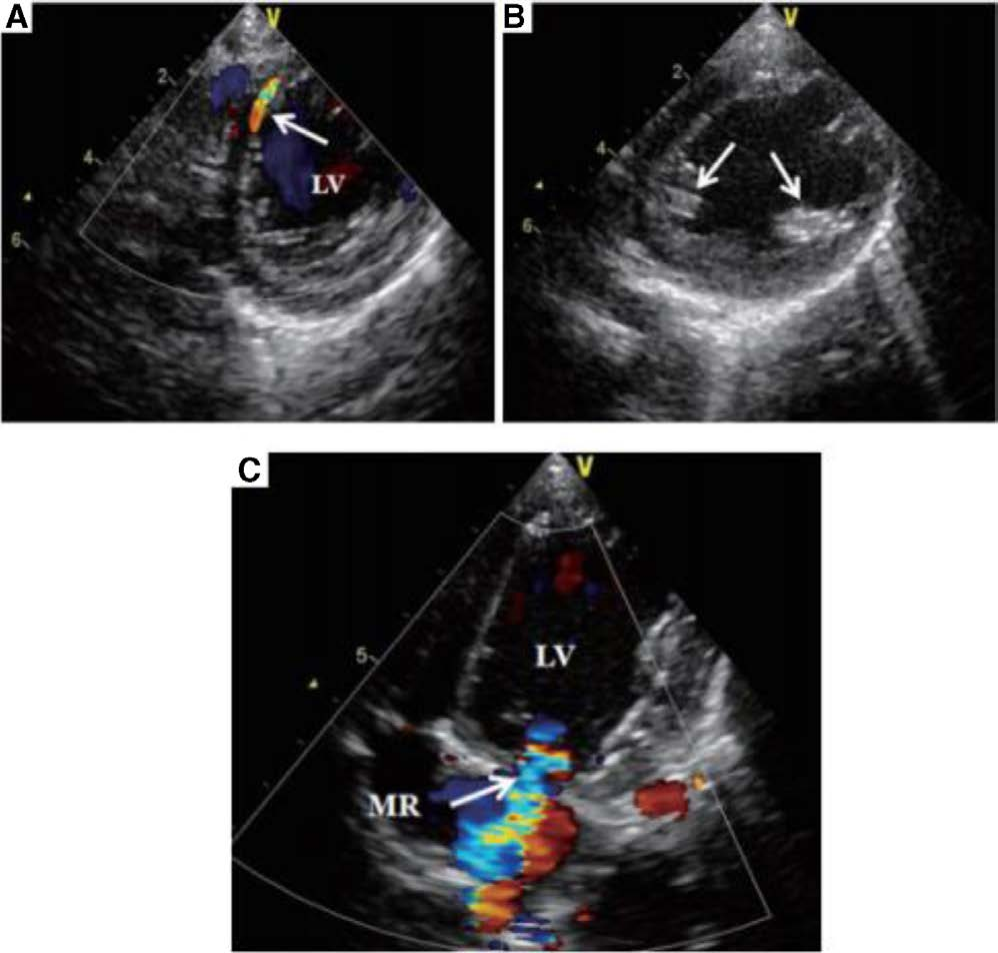
(A) In the parasternal long axis section, color Doppler imaging showed multiple coronary artery collateral branches in the interventricular septum and free ventricular wall (arrow); (B) parasternal short-axis echocardiography showed anechoic enhancement of papillary muscle (arrow); (C) apical 4-chamber echocardiography showed mild mitral regurgitation (arrow).^[7]^

In recent years, the accuracy of echocardiography for the diagnosis of ALCAPA has gradually improved. Conventional echocardiography shows that the LCA originates from the root of the PA and the diastolic blood flow from the LCA to the PA (Fig. 7). Color Doppler shows disordered color flow areas (intercoronary collateral branches) in the interventricular septum and free ventricular wall.^[8]^ YiYu et al analyzed the echocardiographic characteristics of 30 patients with ALCAPA, including 10 adult patients, and found that compared with the infantile type, adult ALCAPA patients were characterized by abundant collateral circulation, no echo enhancement of papillary muscle, no abnormal wall motion, normal or mildly dilated left ventricular diameter, normal ejection fraction, mild mitral regurgitation, and a significantly increased ratio of the RCA to the aortic annulus (> 0.20)^[7]^ (Fig. 8).

**Figure 9.**
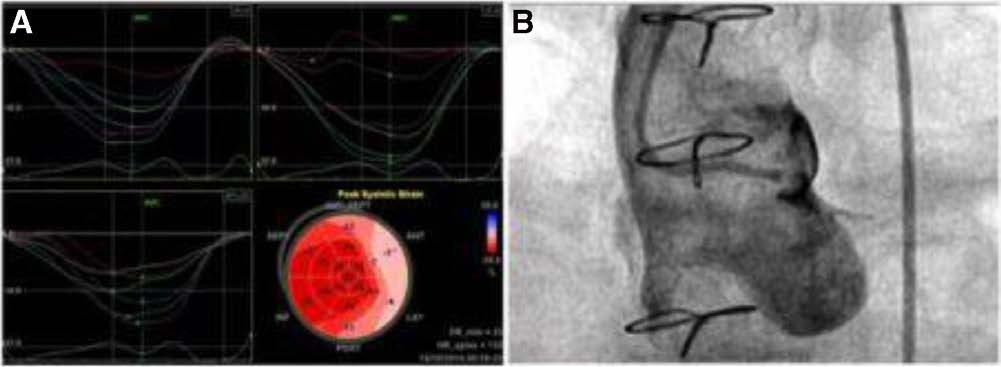
Speckle tracking: (A) The longitudinal strain showed that the longitudinal function of the LCA’s territory was severely impaired. (B) CAG confirmed complete occlusion of the LCA (figure right).^[9]^ CAG = coronary angiography, LCA = left coronary artery.

Conventional echocardiography showed that the left ventricular ejection fraction and other related parameters were normalized after double coronary system reconstruction, but related studies showed that such patients still had residual coronary artery disease or myocardial fibrosis after surgery ^[9]^ (Fig. 9). A new speckle tracking technique identifies persistent cardiac dysfunction by measuring local myocardial strain. Dąbrowska-KugackaIet al have followed 18 patients after ALCAPA for 17 years. The results show that longitudinal myocardial strain damage is not limited to the myocardium in the perfusion area of the LCA but also exists in the RCA area. It can be seen that all cardiac chambers have a certain degree of myocardial injury after operation, which can be used as a potential substrate for malignant arrhythmias and cause of SCD. Subclinical myocardial dysfunction can be identified early using more elaborate echocardiography.^[10]^

**Figure 10.**
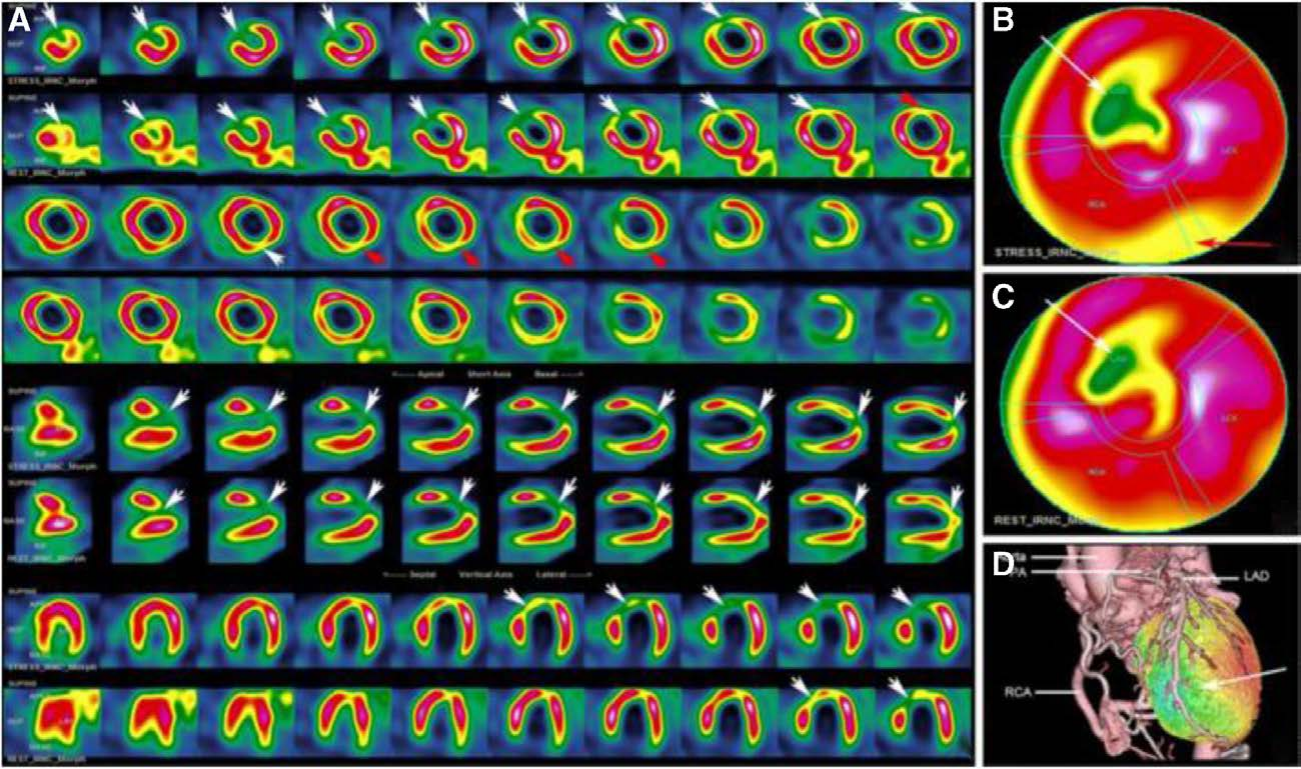
SPECT MPI and fusion images (A) Stress and rest serial images, stress images showed in odd rows and rest images showed in even rows; (B) stress polar map of MPI; (C) rest polar map of MPI; (D) CCTA and surface 3 dimension of stress MPI fused image. SPECT MPI showed fixed perfusion defects in the part of apical segment, apical and middle segments of the anterior wall, and the apical segment of anteroseptal wall (A, B, C and D, as indicated by the white arrows), with a small area of reversible defect in the inferior wall (A and B, as indicated by the red arrows).^[11]^

In addition, when combined with single-photon emission computed tomography myocardial perfusion imaging (SPECT MPI), left ventricular myocyte necrosis, injury, and functional ischemia can be identified early in asymptomatic adults. Chu et al performed SPECT MPI examination in an asymptomatic adult patient with ALCAPA. The results showed an irreversible perfusion defect in the left anterior descending branch (LAD) and a small reversible perfusion defect in the RCA supply area. The formation of a myocardial scar in the anterior wall and myocardial ischemia in the inferior wall have been suggested. (Fig. 10) These patients are at a high risk of ventricular arrhythmia and SCD; therefore, timely surgical intervention is particularly important.^[11]^

Both the 2018AHA/ACC and 2020ESC guidelines for congenital heart disease indicate that once ALCAPA is diagnosed, with or without symptoms, surgery should be performed as soon as possible to restore double coronary artery circulation (Class I),^[12,13]^. The main surgical procedures include coronary artery transplantation and reconstruction, Takeuchi procedure, and ligation of the malformed LCA combined with great saphenous vein or internal mammary artery bypass etc.^[14]^

Early operation and restoration of double coronary blood flow can normalize left ventricular size and function, which reduces mitral regurgitation; however, chronic myocardial ischemic changes such as segmental wall motion abnormalities, perfusion defects, and myocardial scars still exist after surgical repair. After 16 years of follow-up, MikiKanoh et al compared the long-term postoperative outcomes of 2 types of ALCAPA patients and found that adult ALCAPA patients had a higher incidence of major adverse cardiovascular events than infantile patients, mainly due to irreversible left ventricular remodeling caused by long-term chronic myocardial ischemia.^[15]^ Prophylactic implantation of an ICD may be an important means of preventing SCD in such patients. Consequently, the ACC/AHA guidelines recommend that patients with a history of ALCAPA surgical repair undergo noninvasive stress imaging every 3 to 5 years for lifelong follow-up assessment.^[16]^

## 4. Conclusions

ALCAPA is a rare congenital coronary artery malformation, the clinical manifestations of adult ALCAPA patients are varied, for this kind of patients, early diagnosis and surgical correction are usually related to a good prognosis; however, chronic myocardial damage will persist regardless of surgical treatment, and such patients should be followed up for life.

## Author contributions

**Conceptualization:** Yanmin Ge.

**Formal analysis:** Xiaohui Hu.

**Software:** Zhihao Liu.

**Supervision:** Jing Zhang.

**Writing – original draft:** Mengyao Niu.

**Writing – review & editing:** Junduo Wu.
